# Association Between Vaccination Awareness Program Exposure and Full Immunization Coverage Among Mothers in Rural Bangladesh: A Comparative Cross‐Sectional Study in Jashore District

**DOI:** 10.1002/hsr2.73445

**Published:** 2026-10-27

**Authors:** Halima Khatun, Rezwan Hossain, Md. Sohel Mia

**Affiliations:** ^1^ International School Capital Medical University Beijing China; ^2^ Department of Physical Education and Sports Science Jashore University of Science and Technology Jashore Bangladesh; ^3^ Department of Nutrition and Food Technology Jashore University of Science and Technology Jashore Bangladesh

**Keywords:** childhood immunization, Expanded Programme on Immunization (EPI), Jashore, maternal education, rural Bangladesh, vaccination awareness program, vaccine hesitancy

## Abstract

**Background and Aims:**

Childhood immunization is a cost‐effective public health intervention, and Bangladesh has achieved South Asia's highest complete immunization rate, yet village‐level effectiveness of local awareness programs remains poorly evaluated. This study evaluated whether exposure to a village‐level vaccination awareness program was independently associated with full immunization coverage among mothers in Jashore District, Bangladesh, alongside other maternal determinants.

**Methods:**

A community‐based, comparative cross‐sectional study surveyed 1,000 mothers of children aged 0–5 years, sampled by convenience from villages with and without an implemented awareness program, between June and August 2025. Bivariate associations with full immunization status were tested using Chi‐square tests and 95% confidence intervals; multivariable logistic regression estimated adjusted odds ratios (AOR) for all predictors simultaneously.

**Results:**

Of 886 mothers with recorded status, 62.1% (95% CI 58.8%–65.2%) were fully immunized. Maternal education showed the strongest association, rising from 53.6% among mothers with no education to 80.9% among graduates (χ^2^ = 52.60, *p *< 0.001); after adjustment, higher secondary (AOR = 3.42) and graduate education (AOR = 3.64) remained significant, and a supplementary linear‐trend model confirmed a significant dose‐response gradient across education levels (AOR = 1.50 per level, 95% CI 1.31–1.72, *p *< 0.001). Awareness‐program exposure showed no association with full immunization, either before (61.9% vs. 62.2%, *p *= 0.98) or after adjustment (AOR = 0.98, *p *= 0.90). Distance to health facilities (43.2%), fear of side effects (30.9%), and husband disapproval (25.9%) were the leading reported barriers. Mothers with unrecorded EPI status were more often less‐educated than those with recorded status (*p *= 0.004), a missingness pattern addressed explicitly in the Discussion.

**Conclusion:**

Maternal education, not awareness‐program exposure, independently predicted full immunization. Program investment should shift from generic exposure toward literacy‐sensitive content, husband‐inclusive counseling, and outreach‐site expansion to help close Bangladesh's remaining immunization gap.

## Highlights

- 62.1% of children (*n* = 886) in rural Jashore, Bangladesh were fully immunized (EPI).
- Maternal education was the strongest independent predictor of immunization (AOR = 3.64; linear‐trend AOR = 1.50 per level, *p *< 0.001).
- Awareness‐program exposure showed no link to full immunization (AOR = 0.98, *p *= 0.90).
- Distance, side‐effect fear, and husband disapproval were the top reported barriers.
- Mothers with unrecorded EPI status were disproportionately less educated, but this missingness was unrelated to program exposure.
- Programs should add literacy‐sensitive content and husband‐inclusive counseling rather than relying on generic awareness exposure alone.

**Abbreviations**
AOR: adjusted odds ratio
BCG: Bacillus Calmette‐Guérin (tuberculosis vaccine)
BDHS: Bangladesh Demographic and Health Survey
BOMSA: Bangladeshi Ovibashi Mohila Sramik Association
CI: confidence interval
DGHS: Directorate General of Health Services
DPT‐HepB‐Hib: Diphtheria‐Pertussis‐Tetanus‐Hepatitis B‐Haemophilus influenzae type b (pentavalent vaccine)
EPI: Expanded Programme on Immunization
MR: Measles‐Rubella vaccine
NGO: Non‐Governmental Organization
PCV: pneumococcal conjugate vaccine
SAGE: Strategic Advisory Group of Experts (WHO)
SD: Standard Deviation
WHO: World Health Organization

## 1 Introduction

Childhood immunization is among the most cost‐effective public health interventions available, estimated to have averted at least 37 million deaths worldwide between 2000 and 2019 alone [1]. Bangladesh's Expanded Programme on Immunization (EPI) has achieved the highest complete childhood immunization rate in South Asia, at 88.2%, ahead of India, Nepal, Pakistan, and Afghanistan [2], building on early gains linked to outreach‐clinic access and community health workers [3]. Yet national averages mask persistent geographic pockets of zero‐dose and under‐immunized children in haor, hilly, char, coastal, and urban‐slum areas, where maternal education, antenatal‐care attendance, and media access remain the strongest determinants [4, 5, 6].

Three broad determinant classes recur across this literature. First, individual capacity—chiefly maternal education and media exposure—consistently predicts full immunization across four decades of Bangladesh Demographic and Health Survey (BDHS) rounds [7, 8, 9, 10]. Second, household gender dynamics shape uptake: in a 92‐study scoping review, women's lack of autonomous decision‐making was the most frequently cited barrier to immunization across Africa and South Asia [11], a pattern corroborated by registry data on 6.2 million Pakistani children [12]. Third, structural access—particularly distance to health facilities — remains a well‐documented obstacle in rural Bangladesh [13], though person‐delivered outreach can shift completion rates where generic messaging cannot [14]. The WHO SAGE Working Group's “3Cs” framework—confidence, complacency, and convenience—offers a unifying lens for these findings [15]; notably, a 2025 systematic review of 33 hesitancy interventions found that behavior change was substantially harder to achieve than perception change, with in‐person formats outperforming passive ones [16].

A critical gap remains. Because the BDHS and similar national surveys sample a single, pooled population, they cannot isolate the effect of a specific, locally implemented awareness program from the secular improvements in coverage that everyone in a national sample shares to varying, unmeasured degrees. Village‐comparison designs are not unprecedented in Bangladesh: the decades‐long Matlab MCH‐FP outreach program demonstrated broad reproductive‐ and child‐health gains in treatment versus comparison villages [17], and a pre‐post intervention package raised EPI coverage in Dhaka's urban slums [18]. Neither, however, isolated a village‐level vaccination‐awareness program specifically, tested its effect independent of maternal education with multivariable adjustment, or used a contemporary, non‐intervention informational program as the exposure of interest—both instead evaluated multi‐service outreach or service‐delivery packages. To the authors' knowledge, no contemporary Bangladeshi study has directly compared villages with and without an implemented vaccination‐awareness program to test whether program exposure predicts full immunization independent of maternal education—a gap made more urgent by the 2024 global measles resurgence, which has underscored how quickly local coverage gaps translate into outbreak risk [19].

We addressed this gap with a community‐based, comparative cross‐sectional study of 1,000 mothers of children aged 0–5 years in Jashore District, Bangladesh, drawn in approximately equal numbers from villages with and without an implemented vaccination awareness program—a routine, community‐clinic‐ and NGO‐delivered package of household counseling visits, group awareness sessions, and printed EPI‐schedule materials, described fully in Section 2.1. This built‐in exposed‐versus‐unexposed design, combined with both bivariate confidence‐interval estimation and multivariable logistic regression, distinguishes the present study from prior determinant‐focused Bangladeshi research conducted on pooled national samples [7, 8, 9, 10] and constitutes its principal novelty.

This study had three specific objectives [1] to describe the proportion of children fully versus partially immunized under the national EPI schedule among mothers in Jashore District [2] to compare vaccination awareness, vaccine‐safety opinion, and full immunization coverage between mothers in villages with and without an implemented awareness program; and [3] to identify the independent correlates of full immunization—including program exposure, maternal education, and general EPI awareness—using multivariable adjustment. Corresponding to these objectives, we specified four testable hypotheses in advance: (H1) village‐level program exposure is independently associated with full immunization after adjustment for maternal education and awareness; (H2) higher maternal education is independently and positively associated with full immunization; (H3) higher general maternal EPI awareness is independently and positively associated with full immunization; and (H4) distance to health facilities, fear of side effects, and spousal disapproval are the most frequently reported barriers among mothers of partially immunized children. All four hypotheses, and the statistical models used to test them, were specified before the data were analyzed; only the supplementary linear‐trend and missing‐data analyses reported in Sections 3.4 and 3.6 were exploratory, motivated by the pattern of results and specified after seeing the primary analysis.

## 2 Materials and Methods

### 2.1 Study Design and Setting

A community‐based, comparative cross‐sectional study was conducted in the rural field site of Jashore District, Khulna Division, Bangladesh, from 10 June 2025 to 25 August 2025. The design compared immunization outcomes between villages where a vaccination awareness program had been implemented and sociodemographically comparable villages where no such program was in place, so that the specific contribution of program exposure could be examined alongside other individual‐level determinants of childhood immunization. The awareness program consisted of routine community‐level activities delivered by community clinic staff and local NGO outreach workers in the exposed villages, including household‐level counseling visits on the benefits and schedule of childhood immunization, group awareness sessions held at community gathering points, and distribution of printed informational materials on the national EPI schedule; villages in the unexposed/comparison group had no such organized program active during the study period, though mothers there could still access routine EPI services and general health information through the same community clinics, television, and NGOs available to the wider sample (Section 2.4).

### 2.2 Participants

Eligible participants were mothers permanently residing in the selected villages who had at least one living child aged 0 to 5 years at the time of data collection, irrespective of the child's immunization status. Mothers who were not permanent residents of the selected villages, who were unable to communicate in Bangla, or who declined to participate were excluded.

### 2.3 Study Size and Sampling

A convenience sample of 1,000 mothers was recruited: approximately half from villages in which the awareness program had been implemented (the exposed group, *n* = 510) and half from comparable villages in which it had not (the unexposed/comparison group, *n* = 490). Within each selected village, data collectors approached eligible mothers at household visits and community gathering points until the target sample for that village was reached. Because sampling was non‐probabilistic, findings should be interpreted as representative of the sampled villages rather than of Jashore District or Bangladesh as a whole.

The target of 1,000 mothers (approximately 500 per group) was set a priori to give adequate precision for the study's primary comparison: detecting an 8–10 percentage‐point difference in full immunization coverage between exposed and unexposed groups, assuming a baseline coverage of approximately 60%–75% (consistent with prior Jashore‐area estimates), two‐sided α = 0.05, and 80% power, which required approximately 480–500 mothers per group under a standard two‐proportion comparison. The achieved sample met this target in both groups (Table 2); the study was not additionally powered for the smaller education‐category contrasts reported in Table 4, which should be interpreted with this in mind.

### 2.4 Variables and Data Sources

Trained field enumerators administered a pre‐tested, structured questionnaire face‐to‐face in Bangla. The instrument was adapted from item formats used in prior WHO and BDHS immunization‐knowledge surveys [2, 7, 9] and was piloted with a small sample of mothers outside the study villages before fieldwork began, with wording revised for clarity based on pilot feedback; because the final instrument used single‐item, directly observable measures (e.g., a Yes/No awareness item, a three‐category opinion item) rather than a multi‐item psychometric scale, formal scale‐validation statistics such as Cronbach's alpha do not apply and are not reported. The instrument captured maternal age; education level (No Education to Graduate); number of children; age of the youngest child; child EPI vaccination status (Fully immunized, Partially immunized, or not recorded), based on the vaccination card where available and maternal recall otherwise; general awareness of the EPI program; primary source of health information (Community Clinic, TV, NGO); exposure to the village‐level vaccination awareness program—the primary exposure of interest; possession of a vaccination (EPI) card; maternal opinion on vaccine safety; and self‐reported barriers to immunization (distance, fear of side effects, husband disagreement).

Operational definition of full immunization status

A child was classified as “Fully immunized” if the vaccination card (or, where unavailable, maternal recall) confirmed receipt of all vaccine doses scheduled under Bangladesh's national EPI schedule for the child's age at interview—BCG at birth; three doses of pentavalent (DPT‐HepB‐Hib) and oral polio vaccine; pneumococcal conjugate vaccine (PCV); and measles‐rubella (MR) vaccine at 9–15 months, as age‐appropriate—and “Partially immunized” if the child was age‐eligible for but missing one or more of these scheduled doses. Because the underlying instrument recorded overall card‐verified status rather than a dose‐by‐dose checklist, antigen‐specific and dose‐timeliness analyses (e.g., zero‐dose *vs.* under‐immunized distinctions) could not be performed; this is noted as a limitation in Section 5.

### 2.5 Statistical Methods

Descriptive statistics characterized the exposed and unexposed groups. Bivariate associations between categorical predictors and child EPI status (Fully vs. Partially immunized) were tested with Pearson's Chi‐square (χ^2^) test, two‐sided, with 95% confidence intervals for group‐level proportions computed using the Wilson score method. A multivariable binary logistic regression model (outcome: 1 = fully immunized, 0 = partially immunized) estimated adjusted odds ratios (AOR) with 95% Wald confidence intervals for all predictors simultaneously; overall model significance was assessed with a likelihood ratio test against the intercept‐only model, and fit was additionally summarized with McFadden's pseudo‐R^2^. As a pre‐specified sensitivity analysis, education was additionally modeled as a single ordinal (linear‐trend) term in place of category dummies, to test for an overall dose‐response gradient. Respondents with unrecorded EPI status (*n* = 114) were excluded from bivariate and multivariable analyses but retained in descriptive statistics; as a post hoc check, we formally compared mothers with and without recorded EPI status on all observed baseline characteristics (age, number of children, education, awareness, and program exposure) to assess whether this exclusion was likely to bias the reported associations (Section 3.6). Significance was set at α = 0.05 (two‐sided) throughout. *p*‐values are reported to three decimal places when below 0.01 and to two decimal places at or above 0.01, following standard reporting conventions; exact values below 0.001 are reported as “< 0.001.” All analyses were performed in Python 3.11 (pandas 2.x, NumPy 1.x, and SciPy 1.x/statsmodels 0.14).

### 2.6 Ethical Considerations

The study was conducted in compliance with the ethical code of the Declaration of Helsinki and its later amendments, and was approved by the Ethical Review Committee of the Bangladeshi Ovibashi Mohila Sramik Association (BOMSA), Dhaka, Bangladesh (Approval Ref. No: BOMSA/ERC/2024/07). Written informed consent was obtained from all participating mothers before data collection, after adequately explaining the aims and conduct of the study. Participation was voluntary, participant anonymity was preserved throughout data collection, management, and reporting, and the study involved no interventions or potentially risky procedures for participants or their children.

## 3 Results

### 3.1 Sample Characteristics

The mean maternal age was 29.1 years (SD = 6.8; range = 18–40). Mothers reported an average of 2.53 children (SD = 1.13), and the mean age of the youngest child was 2.51 years (SD = 1.43). Regarding education, 35.7% of mothers had completed primary education, 25.3% secondary education, 14.8% had no formal education, 14.4% had higher secondary education, and 9.8% were graduates. Table 1 summarizes the sample's socio‐demographic and informational characteristics.

**Table 1:** Socio‐demographic and informational characteristics of respondents (*N* = 1,000).

Characteristic	Category	*n*	%
Maternal age (years)	Mean 29.1, SD 6.8, range 18–40	1,000	100.0
Education level	No education	148	14.8
	Primary	357	35.7
	Secondary	253	25.3
	Higher secondary	144	14.4
	Graduate	98	9.8
Number of children	Mean 2.53, SD 1.13, range 1–4	1,000	100.0
General EPI awareness	Yes	717	71.7
	No	283	28.3
Village awareness‐program exposure	Yes (exposed)	510	51.0
	No (unexposed)	490	49.0
Vaccination (EPI) card	Yes	817	81.7
	No	183	18.3
Vaccine‐safety opinion	Safe	644	64.4
	Don't Know	215	21.5
	Unsafe	141	14.1

Of the 1,000 mothers, child EPI vaccination status was recorded for 886 (88.6%): 550 (62.1% of those with recorded status; 95% CI [58.8%, 65.2%]) were fully immunized and 336 (37.9%) were partially immunized; status was not recorded for the remaining 114 mothers (11.4%) (see Section 3.6 for a formal comparison of this group against the analytic sample). Among the 886 mothers with recorded EPI status, 743 (83.9%) also named a primary source of health information: 378 (50.9%) cited Community Clinics, 194 (26.1%) cited television, and 171 (23.0%) cited NGOs; source of information was not recorded for the remaining 143 mothers in this subgroup (16.1%).

Because the exposed and unexposed groups were not randomly assigned (Section 2.3), we checked whether they were balanced on observed baseline characteristics before comparing outcomes between them. As shown in Table 2, the two groups did not differ significantly in maternal age, number of children, or education‐level distribution, supporting the interpretation that any subsequent difference (or lack thereof) in immunization outcomes between groups is not attributable to gross baseline imbalance on these measured characteristics.

**Table 2:** Baseline comparability of the awareness‐program (exposed) and comparison (unexposed) groups (*N* = 1,000).

Characteristic	Exposed (*n* = 510)	Unexposed (*n* = 490)	Test statistic	*p*
Maternal age, mean ± SD	29.0 ± 6.8	29.1 ± 6.8	t = −0.25	0.80
Number of children, mean ± SD	2.53 ± 1.13	2.53 ± 1.13	t = −0.02	0.99
Education level, distribution	see Table 1 pattern	see Table 1 pattern	χ2 = 4.04 (4)	0.40

### 3.2 Bivariate Associations With EPI Status

Table 3 reports the percentage of children fully immunized within each category of the study's predictor variables, with 95% confidence intervals (Wilson score method), alongside Pearson chi‐square (χ^2^) test results for the overall association between each predictor and EPI status (Fully vs. Partially immunized, *n* = 886). Full immunization was 65.3% (95% CI [61.5%, 68.8%]) among mothers reporting general EPI awareness versus 53.8% (95% CI [47.6%, 60.0%]) among those without (χ^2^ = 9.38, df = 1, *p *= 0.002) (Figure 1).

**Table 3:** Full immunization coverage by predictor category, with 95% confidence intervals and bivariate chi‐square test results (*n* = 886).

Predictor	Category	*n*	Fully immunized, *n* (%)	95% CI	χ^2^ (df)	*p*
Education level	No Education	125	67 (53.6)	44.9–62.1	52.60 (4)	< 0.001
	Primary	304	154 (50.7)	45.1–56.2		
	Secondary	230	147 (63.9)	57.5–69.8		
	Higher Secondary	133	106 (79.7)	72.1–85.7		
	Graduate	94	76 (80.9)	71.7–87.5		
General EPI awareness	Yes	639	417 (65.3)	61.5–68.8	9.38 (1)	0.002
	No	247	133 (53.8)	47.6–60.0		
Village awareness‐program exposure	Yes (exposed)	449	278 (61.9)	57.3–66.3	0.001 (1)	0.98
	No (unexposed)	437	272 (62.2)	57.6–66.7		
Vaccine‐safety opinion	Safe	572	371 (64.9)	60.9–68.7	5.43 (2)	0.07
	Don't Know	194	112 (57.7)	50.7–64.5		
	Unsafe	120	67 (55.8)	46.9–64.4		
Source of information	Community Clinic	378	232 (61.4)	56.4–66.1	2.08 (2)	0.35
	TV	194	118 (60.8)	53.8–67.4		
	NGO	171	115 (67.3)	59.9–73.8		
Vaccination card possession	Yes	723	451 (62.4)	58.8–65.8	0.091 (1)	0.76
	No	163	99 (60.7)	53.1–67.9		

*Note:* 95% CIs computed using the Wilson score method for a binomial proportion. The χ2, df, and *p*‐value shown for each predictor test the overall association across all of its categories and are reported once, on the first row of each predictor block. Source of information is analyzed among the *n* = 743 mothers with a recorded information source (missing for 143 of the 886).

**Figure 1:**
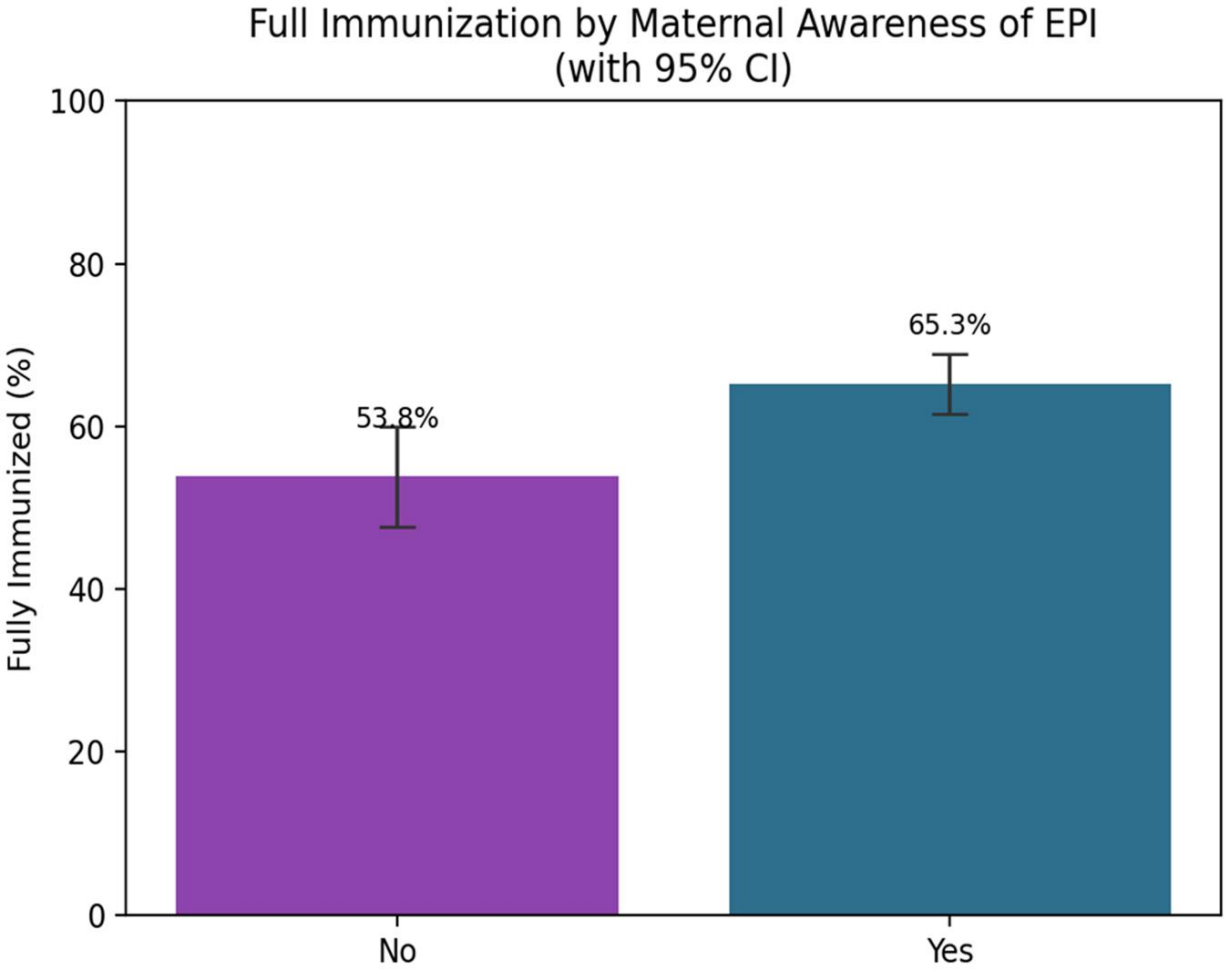
Full immunization rate (%) by maternal awareness of the EPI program, with 95% confidence intervals (*n* = 886).

Full immunization ranged from 53.6% (95% CI [44.9%, 62.1%]) among mothers with no formal education to 80.9% (95% CI [71.7%, 87.5%]) among mothers with graduate‐level education (χ^2^ = 52.60, df = 4, *p *< 0.001) (Figure 2). Full immunization was 61.9% (95% CI [57.3%, 66.3%]) among children in program‐exposed villages versus 62.2% (95% CI [57.6%, 66.7%]) in unexposed villages, a difference of 0.3 percentage points (χ^2^ = 0.001, df = 1, *p *= 0.98). Vaccine‐safety opinion (χ^2^ = 5.43, df = 2, *p *= 0.07), source of information (χ^2^ = 2.08, df = 2, *p *= 0.35), and vaccination‐card possession (χ^2^ = 0.091, df = 1, *p *= 0.76) showed no statistically significant association with EPI status at α = 0.05.

**Figure 2:**
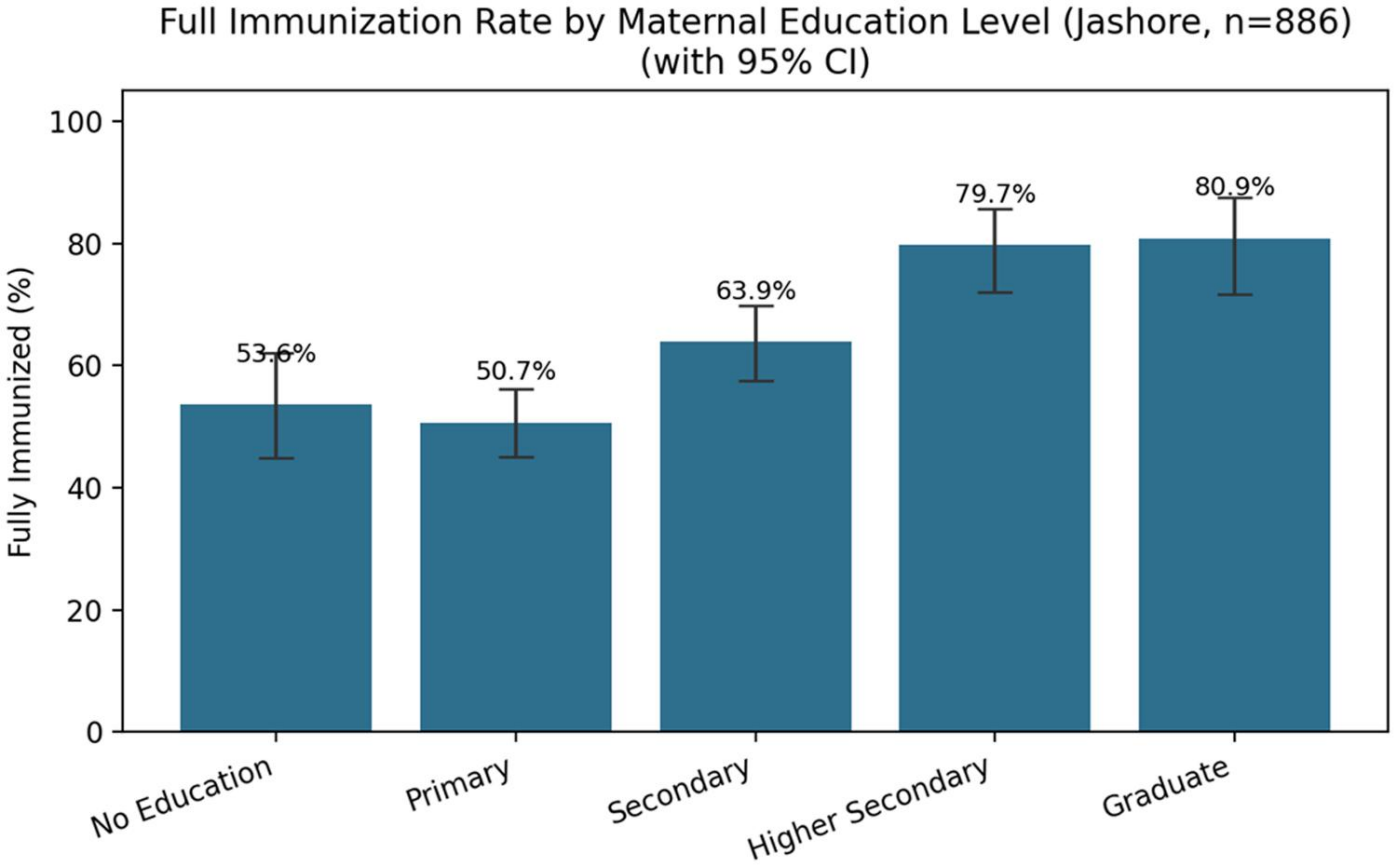
Full immunization rate (%) by maternal education level (*N* = 886 with recorded EPI status).

Mean maternal age was 29.14 years (SD = 6.85) among mothers of fully immunized children and 29.01 years (SD = 6.73) among mothers of partially immunized children. Mean number of children was 2.51 (SD = 1.10) among fully immunized cases and 2.57 (SD = 1.18) among partially immunized cases.

### 3.3 Multivariable Logistic Regression

A multivariable logistic regression model was fitted to estimate the independent association of each predictor with full immunization status while adjusting for all other variables simultaneously (*n* = 886). Predictors entered were maternal age, number of children, education level (reference: No Education), general EPI awareness (reference: No), village awareness‐program exposure (reference: No/unexposed), vaccination‐card possession (reference: No), vaccine‐safety opinion (reference: Don't Know), and source of information (reference: Community Clinic; a missing‐source indicator was included to retain the full sample). The overall model was statistically significant relative to the intercept‐only model (likelihood ratio χ^2^ [13] = 59.98, *p *< 0.001; McFadden pseudo‐R^2^ = 0.051). Table 4 reports adjusted odds ratios (AOR), 95% confidence intervals, and Wald‐test p‐values for each predictor.

**Table 4:** Multivariable logistic regression predicting full immunization status (*n* = 886).

Predictor	AOR	95% CI	*p*
Maternal age (per year)	1.00	0.98–1.02	0.84
Number of children	0.95	0.83–1.07	0.40
Education: Primary (ref: No Education)	0.87	0.57–1.33	0.52
Education: Secondary (ref: No Education)	1.53	0.96–2.43	0.07
Education: Higher Secondary (ref: No Education)	3.42	1.94–6.04	< 0.001
Education: Graduate (ref: No Education)	3.64	1.92–6.92	< 0.001
General EPI awareness: Yes (ref: No)	0.90	0.62–1.32	0.59
Village awareness‐program exposure: Yes (ref: No)	0.98	0.74–1.30	0.90
Vaccination card: Yes (ref: No)	1.12	0.78–1.61	0.54
Vaccine‐safety opinion: Safe (ref: Don't Know)	1.14	0.79–1.64	0.49
Vaccine‐safety opinion: Unsafe (ref: Don't Know)	0.86	0.53–1.39	0.55
Source of information: TV (ref: Community Clinic)	1.03	0.71–1.49	0.87
Source of information: NGO (ref: Community Clinic)	1.28	0.86–1.90	0.22

*Note:* AOR = adjusted odds ratio from a single multivariable logistic regression model including all listed predictors simultaneously. CI = confidence interval. Model additionally adjusted for a missing‐source‐of‐information indicator (not shown) to retain respondents with no recorded information source.

In the adjusted model, higher secondary education (AOR = 3.42, 95% CI [1.94, 6.04], *p *< 0.001) and graduate‐level education (AOR = 3.64, 95% CI [1.92, 6.92], *p *< 0.001) remained significantly associated with full immunization relative to no formal education. Secondary education was not statistically significant after adjustment (AOR = 1.53, 95% CI [0.96, 2.43], *p *= 0.07). General EPI awareness (AOR = 0.90, 95% CI [0.62, 1.32], *p *= 0.59) and village awareness‐program exposure (AOR = 0.98, 95% CI [0.74, 1.30], *p *= 0.90) were not statistically significant predictors of full immunization after adjustment for education and the other covariates in the model. No other predictor reached statistical significance at α = 0.05 in the adjusted model.

### 3.4 Sensitivity Analysis: Linear Trend Across Education Levels

Because the categorical education contrasts in Table 4 leave the pattern for intermediate categories (Primary, Secondary) statistically ambiguous, we re‐fitted the multivariable model with education entered as a single ordinal (0–4) linear‐trend term in place of the four category dummies, adjusting for the same covariates. This confirmed a strong, statistically significant overall dose‐response gradient: each one‐category increase in maternal education was associated with 50% greater adjusted odds of full immunization (AOR = 1.50, 95% CI [1.31, 1.72], *p *< 0.001). This trend‐based result is consistent with, and reinforces, the categorical finding in Table 4 that education—rather than awareness, program exposure, or any other measured variable—is the dominant independent correlate of full immunization in this sample, while the non‐significant Primary and Secondary category contrasts most likely reflect reduced precision within individual strata rather than a genuine departure from a monotonic education gradient.

### 3.5 Maternal Opinion on Vaccine Safety

Overall, 644 mothers (64.4%) considered vaccines safe, 215 (21.5%) reported not knowing, and 141 (14.1%) considered vaccines unsafe (Figure 3). By education level, 56.1% of mothers with no formal education and 75.5% of graduate mothers reported that vaccines were safe; 26.4% of mothers with no formal education and 15.3% of graduate mothers reported not knowing.

**Figure 3:**
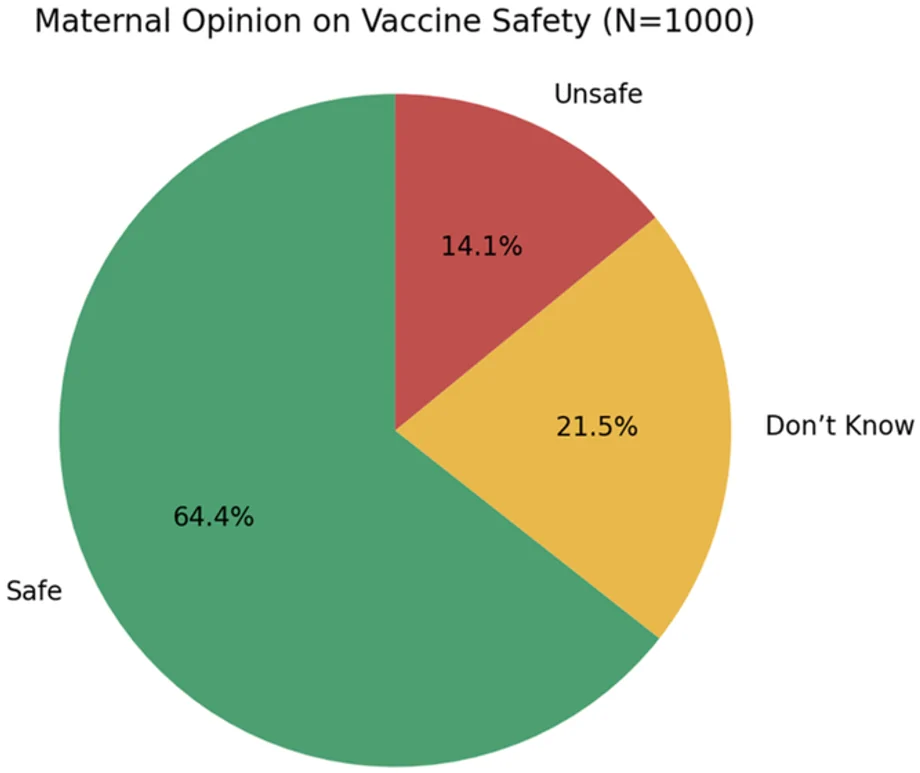
Maternal opinion on vaccine safety (*N* = 1,000).

### 3.6 Comparison of Mothers With and Without Recorded EPI Status

Because 114 mothers (11.4%) lacked a recorded EPI status and were excluded from the bivariate and multivariable analyses above, we compared this excluded group against the 886 mothers with recorded status on all observed baseline characteristics, to assess whether the exclusion was likely to bias the reported associations. Mothers with unrecorded status did not differ significantly from those with recorded status in age (29.0 vs. 29.1 years, *p *= 0.93), number of children (2.46 vs. 2.54, *p *= 0.53), village awareness‐program exposure (χ^2^ = 0.22, df = 1, *p *= 0.64), or general EPI awareness (χ^2^ = 0.51, df = 1, *p *= 0.47). However, they differed significantly by education level (χ^2^ = 15.26, df = 4, *p *= 0.004): mothers with no formal education were over‐represented among those with unrecorded status (23/148, 15.5%) relative to graduates (4/98, 4.1%). This indicates that missingness in EPI status was not completely random but was systematically associated with maternal education—the study's key correlate of full immunization—while unrelated to program exposure or awareness. We discuss the implications of this pattern for interpreting the study's principal findings in Section 5.

### 3.7 Barriers to Immunization

Of the 1,000 mothers surveyed, 440 (44.0%) reported at least one specific barrier to immunization; the remaining 560 (56.0%) reported no barrier or did not specify one. Among the 440 reporting a barrier, distance to the health facility was most common (190; 43.2%), followed by fear of side effects (136; 30.9%) and disapproval from the husband (114; 25.9%) (Figure 4). Among mothers of partially immunized children who reported a barrier, the corresponding figures were distance 43.9%, fear 28.0%, and husband disagreement 27.4%.

**Figure 4:**
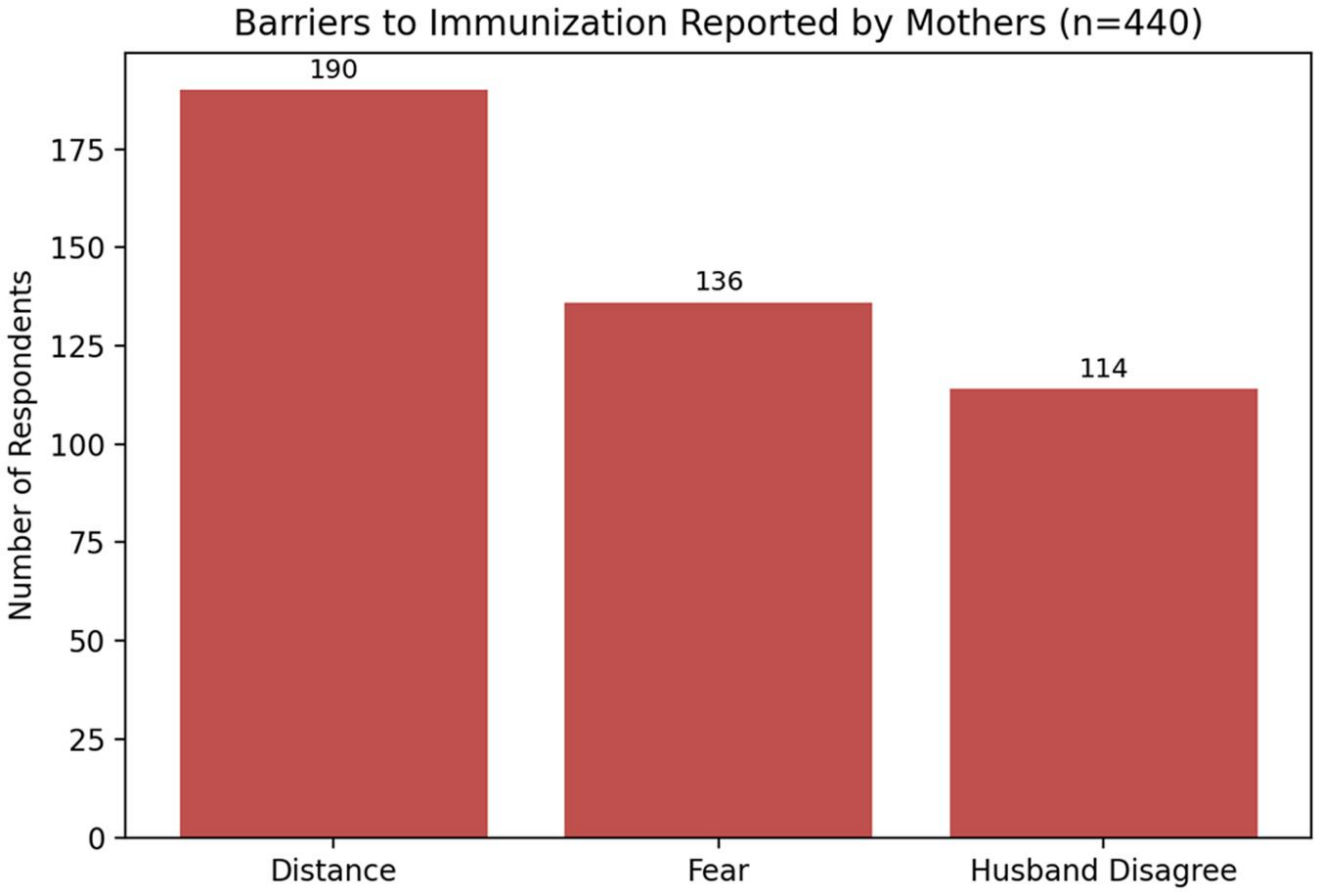
Barriers to immunization reported by mothers who cited at least one barrier (*n* = 440).

## 4 Discussion

This community‐based, comparative study evaluated whether a village‐level vaccination awareness program was independently associated with full childhood immunization in rural Jashore District, Bangladesh, using a design that distinguished mothers in program‐exposed villages from mothers in matched, unexposed villages.

### 4.1 Principal Findings

Four results anchor this study's interpretation. First, maternal education was by far the strongest correlate of full immunization, rising from 53.6% among mothers with no education to 80.9% among graduates (χ^2^ = 52.60, *p *< 0.001), remaining independently significant after adjustment for higher secondary (AOR = 3.42) and graduate (AOR = 3.64) education, and confirmed as a significant overall dose‐response gradient in the linear‐trend sensitivity analysis (AOR = 1.50 per level, *p *< 0.001; Section 3.4). The categorical gradient was non‐monotonic at lower levels: primary education showed marginally lower adjusted odds than no formal education (AOR = 0.87, 95% CI [0.57, 1.33], *p *= 0.52), though neither differed significantly from the reference category, and the overall linear trend across all five categories remained highly significant. This dip most plausibly reflects sampling variability within a modestly sized stratum rather than a true protective effect of no schooling, since its wide confidence interval spans 1 and the corresponding bivariate rate (50.7%) was likewise the lowest of any education category; it is noted here as a pattern for confirmation in larger, probability‐based samples rather than substantive interpretation. Second, general EPI awareness predicted full immunization in bivariate analysis but not after adjustment, and formal exposure to the village awareness program was not significant at any stage of analysis (AOR = 0.98, *p *= 0.90). Third, distance to a health facility, fear of side effects, and husband disapproval emerged as the dominant self‐reported barriers, together cited by 44.0% of mothers. Fourth, mothers with unrecorded EPI status were disproportionately less educated than mothers with recorded status (Section 3.6), a pattern that does not change the study's principal conclusions but should be weighed when generalizing the reported coverage estimate. Against the pre‐specified hypotheses, H1 (independent program‐exposure effect) was not supported; H2 (independent education effect) was supported for higher secondary and graduate education, and further reinforced by the significant overall linear trend; H3 (independent awareness effect) was supported only in unadjusted analysis, indicating substantial confounding by education; and H4 (distance, fear, and spousal disapproval as leading barriers) was supported.

### 4.2 Comparison With Existing Literature

The education gradient observed here replicates national‐level evidence: using the 2017–18 BDHS [8, 9] both found markedly lower vaccination completion among children of uneducated or primary‐educated mothers, and [10] reported comparable individual‐ and community‐level education effects in a pooled multilevel analysis. This pattern is further corroborated by two recent multi‐country South Asian analyses: a six‐country study identifying maternal education and household wealth quintile as consistent predictors of full vaccination coverage across Afghanistan, Bangladesh, India, Nepal, and Pakistan [20], and a South Asia‐wide analysis of measles‐vaccine dosing that similarly found parental characteristics, media access, and antenatal‐care attendance—factors closely related to maternal education and health‐system contact—to be the key determinants of complete two‐dose measles coverage [21]. Together, these studies reinforce that the education gradient observed in this Jashore sample is not a local anomaly but part of a broader regional pattern. The barrier findings likewise replicate earlier work [3, 13] both identified facility distance as a significant predictor of complete immunization in rural Bangladesh, and an [11] 92‐study scoping review identified women's lack of decision‐making autonomy—mirrored here by the husband‐disapproval barrier—as the most frequently documented gender obstacle to immunization across Africa and South Asia.

### 4.3 Explaining the Null Program‐Exposure Finding

The absence of an awareness‐program effect, though unanticipated, is plausible on three grounds. First, informational diffusion may have equalized exposure: mothers in “unexposed” villages drew on the same community clinics, television, and NGOs as the sample overall, which could dilute any incremental program effect. Second, per the WHO SAGE 3Cs framework, an intervention that raises confidence or complacency will not move coverage if convenience barriers—distance, spousal disapproval—remain binding [15]. Third, the attenuation of the awareness‐immunization association after adjusting for education suggests that awareness functions largely as a downstream marker of education rather than an independent causal driver—consistent with the education gradient in vaccine‐safety perception (56.1% of mothers with no formal education considered vaccines safe, *vs.* 75.5% of graduates, with the gap concentrated in “don't know” responses).

### 4.4 Implications of the Missing‐Data Pattern

The finding that unrecorded EPI status was concentrated among less‐educated mothers (Section 3.6) has two implications. Practically, it suggests that record‐keeping and card retention are themselves weaker in the same low‐education households that already show the lowest immunization completion, meaning the true coverage gap by education may be even larger than the 53.6%–80.9% range observed among mothers with verifiable records. Analytically, because missingness was unrelated to program exposure or awareness, the study's central finding—a null program effect—is unlikely to be an artifact of this missingness; a null result cannot easily be manufactured by selectively losing cases on a variable (education) that is itself unrelated to the group being compared (exposed vs. unexposed). We nonetheless treat the 62.1% coverage estimate as applicable to mothers with verifiable records rather than to the full sample, consistent with its stated denominator throughout.

### 4.5 Implications and Future Research

Practically, program monitoring in Jashore should shift from attendance counts to completed‐dose outcomes, pairing awareness content with expanded outreach‐site density, mobile or door‐to‐door services, and explicit side‐effect counseling. Theoretically, the attenuation of the awareness effect after adjustment supports treating awareness as a mediator or marker of maternal education within the 3Cs framework rather than as an independent confidence‐building lever. For policy, the Directorate General of Health Services (DGHS) and its NGO partners may achieve larger coverage gains by reallocating awareness‐program budgets toward literacy‐sensitive content, husband‐inclusive counseling, and outreach‐site expansion in high‐distance areas—consistent with geo‐pocket targeting already used to prioritize under‐immunized communities in Bangladesh [4, 5] and with propensity‐matched evidence that structured, person‐delivered outreach improves timely vaccination completion [14]—supporting progress toward the Immunization Agenda 2030 target of halving zero‐dose and under‐immunized children. Future research should prioritize a cluster‐randomized or stepped‐wedge trial to establish the program's causal effect, longitudinal dose‐by‐dose follow‐up to capture timeliness and dropout, mixed‐methods process evaluation to clarify mechanisms, formal mediation analysis of education's pathway through awareness, inclusion of household wealth and antenatal‐care variables, improved record‐keeping to reduce the education‐linked missingness identified in Section 3.6, and replication across multiple districts using probability‐based sampling.

## 5 Limitations

Several limitations should be considered when interpreting these findings. First, villages were not randomly assigned to program and comparison status, and participants within villages were recruited by convenience rather than probability sampling; pre‐existing differences between exposed and unexposed villages (e.g., in health‐worker staffing or baseline literacy) could confound the observed lack of association between program exposure and immunization status, and results may not be representative of Jashore District or Bangladesh as a whole. Second, the cross‐sectional design precludes causal inference; associations between education, awareness, and immunization status may partly reflect unmeasured socio‐demographic confounders that this survey did not capture—most notably household wealth or income, but also maternal age at marriage, family structure (nuclear *vs.* joint household), and the household head's occupation, each of which prior Bangladeshi studies have linked to immunization uptake and which future surveys in this setting should collect. Third, EPI status, awareness, and barriers were self‐reported by mothers and may be subject to recall or social‐desirability bias, particularly for sensitive items such as husband disapproval. Fourth, EPI status was not recorded for 11.4% of respondents; as shown in Section 3.6, these mothers were disproportionately less educated than the analytic sample, so the reported 62.1% coverage figure should be understood as applying to mothers with verifiable records rather than as an unbiased estimate for the full sampled population—though, because this missingness was unrelated to program exposure, it is unlikely to explain the study's null program‐exposure finding. Fifth, the dataset records overall card‐verified or recalled EPI status rather than a dose‐by‐dose checklist, so it does not distinguish between specific antigens (e.g., BCG, pentavalent, measles‐rubella) or dose timeliness, limiting comparability with BDHS‐based studies that model complete eight‐dose schedules and precluding zero‐dose versus under‐immunized distinctions. Sixth, several of the education‐category confidence intervals in Table 4 are relatively wide (e.g., Higher Secondary: 95% CI [1.94, 6.04]; Graduate: 95% CI [1.92, 6.92]), reflecting the more modest size of these subgroups (*n* = 133 and *n* = 94, respectively) rather than a precisely estimated effect; with 14 predictor terms fitted on *n* = 886 observations, some degree of overfitting cannot be excluded, and these specific point estimates should be interpreted as indicative rather than precise until replicated in a larger sample — the linear‐trend sensitivity analysis (Section 3.4), which uses a single, more stable degree of freedom for education, is offered partly as a check against this risk and yielded a consistent conclusion. Finally, as a single rural field‐site study, findings from Jashore may not generalize to other unions, districts, or divisions of Bangladesh with different accessibility, ethnic, or religious profiles.

### 5.1 Strengths and Limitations of This Study

- This is one of the first studies in Bangladesh to compare childhood immunization outcomes between villages with and without an implemented vaccination awareness program, rather than relying on pooled, undifferentiated survey data.
- The analysis combines bivariate testing with 95% confidence intervals, a multivariable logistic regression, and a pre‐specified linear‐trend sensitivity analysis, allowing independent effects to be distinguished from confounded bivariate associations.
- A relatively large sample (*N* = 1,000) allowed reasonably precise confidence intervals even for smaller subgroups, and a formal missing‐data comparison (Section 3.6) directly addresses whether excluded cases could explain the study's principal findings.
- Villages were not randomly assigned to program status and participants were recruited by convenience sampling, so causal effectiveness of the awareness program cannot be established and residual confounding cannot be excluded.
- The cross‐sectional design, reliance on maternal self‐report, and absence of household wealth and antigen‐specific data further limit causal and comparative interpretation (see Section 5).

## 6 Conclusion

In this rural Jashore field site, 62.1% of children with recorded status were fully immunized under the national EPI schedule. Contrary to expectation, exposure to a village‐level vaccination awareness program showed no independent association with full immunization, whereas maternal education was the only variable independently predictive of completion after multivariable adjustment—a finding reinforced by a significant linear dose‐response trend across education levels; distance to health facilities, fear of side effects, and husband disapproval remained the dominant reported barriers. A supplementary analysis further showed that missingness in the immunization outcome was concentrated among less‐educated mothers but unrelated to program exposure, indicating that the null program finding is unlikely to be a missing‐data artifact. These findings indicate that, in comparable rural Bangladeshi settings, immunization completion is driven more by maternal education and structural/social access than by generic awareness exposure. Program design should therefore prioritize literacy‐sensitive, actionable content for the least‐educated mothers, husband‐inclusive counseling, expanded outreach‐site density in high‐distance areas, and completed‐dose—rather than attendance‐based—monitoring. Confirming these findings causally will require cluster‐randomized trials and longitudinal dose‐tracking beyond the present cross‐sectional, convenience‐sampled design, but the results already offer actionable, village‐level evidence to help close Bangladesh's remaining immunization gap and support progress toward the Immunization Agenda 2030 goal.

## Author Contributions

**Halima Khatun:** conceptualization, methodology, supervision, formal analysis, writing – review and editing, writing – original draft. **Rezwan Hossain:** methodology, visualization, writing – original draft, writing – review and editing, formal analysis, software, data curation. **Md. Sohel Mia:** investigation, data curation, validation, conceptualization.

## Funding

This research did not receive any specific grant from funding agencies in the public, commercial, or not‐for‐profit sectors. As there was no external funder, no external party had any role in the design of the study, the collection, analysis, or interpretation of the data, the writing of the manuscript, or the decision to submit the results for publication.

## Ethics Statement

The study was conducted in compliance with the ethical code of the Declaration of Helsinki and its later amendments and was approved by the Ethical Review Committee of the Bangladeshi Ovibashi Mohila Sramik Association (BOMSA), Dhaka, Bangladesh (Approval Ref. No: BOMSA/ERC/2024/07). Written informed consent was obtained from all participating mothers prior to data collection.

## Consent

Not applicable. This manuscript contains no individual person's data in any form (no images, videos, or identifiable case details).

## Conflicts of Interest

The authors declare no conflicts of interest.

## Declaration of Generative AI and AI‐Assisted Technologies

The authors declare that generative artificial intelligence (AI) tools, specifically **claude**, were utilized solely for language refinement, formatting, and grammatical corrections during the manuscript preparation. The authors confirm that all scientific concepts, data analysis, and conclusions are entirely their own work. The authors take full responsibility for the final published content.

## Reference Verification Statement

All references cited in this manuscript have been individually checked against PubMed's retraction tags and the Retraction Watch database at the time of submission; none were identified as retracted or subject to a published correction affecting the content cited here. This verification process also identified and corrected several volume/page or article‐number inaccuracies in the reference list relative to the original publications, which have been updated accordingly.

## Transparency Statement

The corresponding author and manuscript guarantor, Rezwan Hossain, affirms that this manuscript is an honest, accurate, and transparent account of the study being reported; that no important aspects of the study have been omitted; and that any discrepancies from the study as originally planned—including the corrections made in response to peer review—have been explained in full in the Methods, Results, and Discussion above.

## Data Availability

The de‐identified dataset analyzed in this study is available from the corresponding author upon reasonable request, subject to the ethical approval terms granted by the Ethical Review Committee of BOMSA (Approval Ref. No: BOMSA/ERC/2024/07).
